# Long-Term Occurrence of Deoxynivalenol in Feed and Feed Raw Materials with a Special Focus on South Korea

**DOI:** 10.3390/toxins10030127

**Published:** 2018-03-16

**Authors:** Juhee Park, Hansub Chang, Dongho Kim, Soohyun Chung, Chan Lee

**Affiliations:** 1 Advanced Food Safety Research Group, BrainKorea21 Plus, Department of Food Science and Technology, Chung-Ang University, 4726, Seodong-daero, Anseong-si 17546, Gyeonggi-do, Korea; bjhwngml@naver.com; 2 National Agricultural Products Quality Management Service, 141, Yongjeon-ro, Gimcheon-si 39660, Gyeongsangbuk-do, Korea; jjhs@korea.kr (H.C.); anoldmu@korea.kr (D.K.); 3 Department of Integrated Biomedical and Life Science, Korea University, Seoul 02841, Korea; chungs59@korea.ac.kr

**Keywords:** mycotoxin, deoxynivalenol, animal feed, compound feed, feed ingredient

## Abstract

The *Fusarium* fungi produce toxic substances called mycotoxins, which can cause disease and harmful effects in grains, livestock, and humans. Deoxynivalenol (DON), also known as vomitoxin, is one of the *Fusarium* mycotoxins that is known to cause vomiting in livestock. This study shows the occurrence of deoxynivalenol in feedstuffs (compound feed and feed ingredients) between 2009 and 2016 in South Korea. A total of 653 domestic samples were collected at five time points, including 494 compound feed samples and 159 feed ingredient samples. DON contamination levels were analyzed using high-performance liquid chromatography (HPLC) with pretreatment using an immunoaffinity column (IAC). The limit of detection (LOD) and the limit of quantification (LOQ) were estimated at 1–10 µg/kg and 3–35 µg/kg, respectively. Two compound feeds (two gestating sow feed samples) out of 160 pig feed samples exceeded the European Commission (EC) guidance value, while no feed ingredient samples exceeded the EC or South Korean guidance values. There were statistically significant differences in the mean contamination levels of compound feed and feed ingredients that indicated a decreasing trend over time.

## 1. Introduction

Mycotoxins produced from *Fusarium* species are a serious problem for grains and maize, and many researchers have reported that *Fusarium* toxins may affect livestock and humans in various countries [1,2]. *Fusarium* toxins have traditionally been associated with the temperature at which the cultivation, harvest, and storage of cereals occur. These fungi are mesophilic with an optimum temperature for growth and mycotoxin production between 20 and 30 °C. Therefore, many reports have demonstrated the global scale of grain contamination with a number of *Fusarium* toxins including fumonisins (FUMs), zearalenone (ZEN), and deoxynivalenol (DON) to name a few [3,4,5].

**Figure 1:**
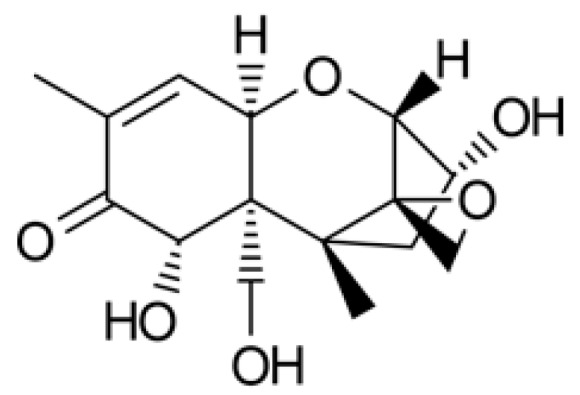
Chemical structure of deoxynivalenol (DON).

Trichothecenes are mycotoxins produced by various *Fusarium* genera that are classified into A, B, C, and D type groups according to their chemical properties. DON belongs to type B [6], which has a double bond with oxygen at the number 8 carbon in the molecular structure [7]. The chemical structure of DON is shown in Figure 1.

DON was discovered by Morooka et al. in 1972 as produced by *Fusarium graminearum* on cereals such as wheat, maize, and barley [8]. DON is a colorless bed-type crystal granule, and has heat stability at 120 °C. DON is soluble in water and some organic solvents [9]. Protein synthesis is inhibited by this mycotoxin through interference with peptidyl transferase activity combined with intracellular ribosomes [10]. DON induces impaired growth and weight gain in animals [11]. In addition, DON causes typical intoxication of livestock and also induces vomiting, leading to a decrease in feed intake and feeding refusal symptoms at high concentrations. In a chronic toxicity test at low concentrations, DON did not induce any changes related to animal behavior; biochemical, hematological, or biochemical characteristics; or immunological parameters [12]. Swine was highly sensitive to DON. This toxin is classified as a group 3 carcinogen by the International Agency for Research on Cancer (IARC) [9].

The occurrence of DON in food and animal feed is a significant problem for the livestock industry and for the supply chain and international trade in food and feed [1,13]. Rodrigues and Naehrer [14] reported a three-year survey related to the presence of mycotoxins in feedstuffs and feed worldwide. Aflatoxins (AFs), ZEN, DON, FUMs, and ochratoxin A (OTA) were detected in 33, 45, 59, 64, and 28% of all analyzed samples including soybean meal, wheat, corn, finished feed samples, and dried distillers grains with solubles (DDGS) from the Americas, Europe, and Asia. DON showed the second highest occurrence.

In China, a total of 56 wheat samples were analyzed for DON contamination levels [15]. Among them, 89.3% of cereal samples were contaminated with DON in the range of 259 to 4975 μg/kg. In a study in Tunisia, durum wheat samples (65) were contaminated with DON [16] that ranged from 12.8 ± 5% to 30.5 ± 13.3% μg/g. Pietsch et al. [17] reported that 81.8% of commercial fish feed samples (11) collected from central Europe were contaminated with DON. The average contamination value was 289 μg/kg feed. In South Korea, DON was detected in 91.3% and 53.3% of compound feeds and feed ingredients, respectively [18].

The European Commission (EC) provided recommended guidance values for mycotoxins in animal feed for aflatoxin (AF), ochratoxin (OCT), and other *Fusarium* mycotoxins. The USA has also managed aflatoxin, DON, and fumonisins (FUM) in feeds [19]. In South Korea, the guidance level for AF and OCT in animal feed has been managed according to the guidelines for livestock and fish feed up to 2014. However, recently, levels of *Fusarium* mycotoxins in feed have been controlled based on continuous monitoring results and the EC’s recommended guidance values. The values for DON management in the EU [19] and South Korea [20] are shown in Table 1.

Animal feedstuffs are composed of grains and grain byproducts with vegetable proteins. In 2012, 15,350 tons of feed ingredients were imported by South Korea from many countries such as China, USA, Europe, Canada, South Africa, South East Asia, Australia, and India [21]. This indicates that not only animal feedstuffs but also feed ingredients including grain, grain by-products, and meal should be controlled for feed safety. Therefore, this study was performed to monitor DON contamination levels in feed ingredients, as well as in animal feedstuffs, over an eight-year period to estimate the tendency of DON contamination.

## 2. Results

### 2.1. Method Validation

**Figure 2:**
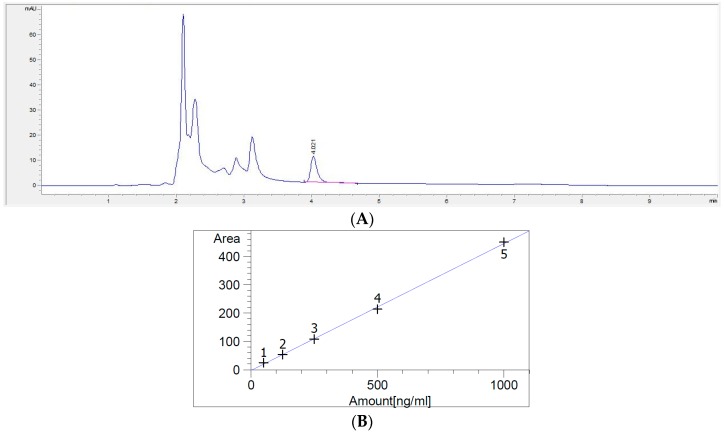
Calibration curve for DON (**A**) and the HPLC chromatogram (**B**).

A clear peak for DON was observed on the HPLC (high-performance liquid chromatography) chromatogram by pretreatment using an immunoaffinity column (IAC) (Figure 2A). The regression coefficient was calculated to be over 0.999 based on the standard curve (solvent-based) for DON (Figure 2B). The limit of detection (LOD) was 1 to 10 μg/kg and the limit of quantification (LOQ) was 3 to 35 μg/kg for tested years. Accuracy was calculated from the average recovery ratio obtained from the recovery test, and precision was the percent relative standard deviation (%RSD) calculated from the same tests. The accuracy range was 83.3% to 108.3%, and the RSD was 1.4% to 13.9% (Table 2). These results meet the EC-recommended guidance value for DON during all test years, meaning that accuracy was 70% to 120% and precision was under 20% [22].

**Figure 3:**
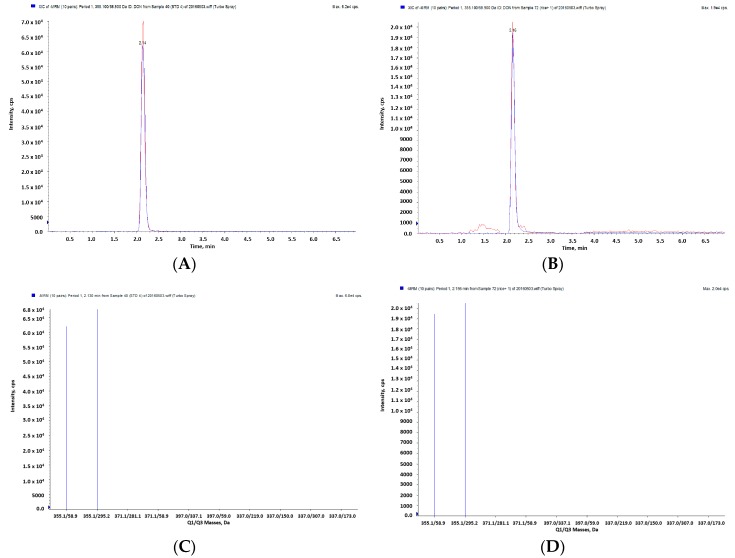
Identification of DON by LC-MS/MS. An extracted ion chromatogram (XIC) of the DON standard at 510 ppb (**A**) and DON in a feed sample (**B**). The ion spectrum (product ion) of standard DON (**C**) and those in a feed sample (**D**).

Further identification of DON was performed using LC-MS/MS with the extracted ion chromatogram (XIC) and mass spectrum (Figure 3). The XIC for DON in standard solution and in contaminated samples (Figure 3A,B) showed the same precursor ion with *m*/*z* of 355.1 ([M − H]^−^). The XIC for DON showed the two product ions of *m*/*z* 58.9 and 295.2 as shown in Figure 3C, and the two product ions from XIC for the feed sample were exactly matched with those of DON in the standard solution (Figure 3D).

### 2.2. Occurrence of DON in Compound Feeds between 2009 and 2016

The DON contamination level was assessed in 174 cattle feed, 160 pig feed, and 160 poultry feed samples (Table 3). Samples were collected in 2009, and in every subsequent two years from 2010 to 2016 (2010, 2012, 2014 and 2016). Most samples (97.7% of cattle feed, 93.1% of pig feed, and 95.0% of poultry feed samples) were contaminated with DON; DON was found in 95.3% of the samples. To describe how were samples with DON level < LOD managed for statistical analysis, statistical definition (Percentage of Left censored results; % LC) was applied to exhibit a data point in statistics below a certain value but it is unknown by how much. % LC was generally used in safety assessment as described by EFSA [23], and we applied this statistics term to explain the percentage of data below LOD which exhibit where the observed data is located in percentage. The mean concentration of DON contamination in all feeds was 374.5 μg/kg in the range of 0.4 to 2420.0 μg/kg. The mean contamination level of DON in cattle feed, pig feed, and poultry feed samples was 646.3, 231.5, and 222.0 μg/kg, respectively. The highest level of DON contamination was observed in cattle feed samples among the three types of compound feed samples after statistical comparison. In a one-way analysis of variance (ANOVA), there was a significant difference among the three groups of compound feed samples (*p* < 0.001). Scheffe’s method was used to determine the average contamination difference in DON (Table 4).

**Figure 4:**
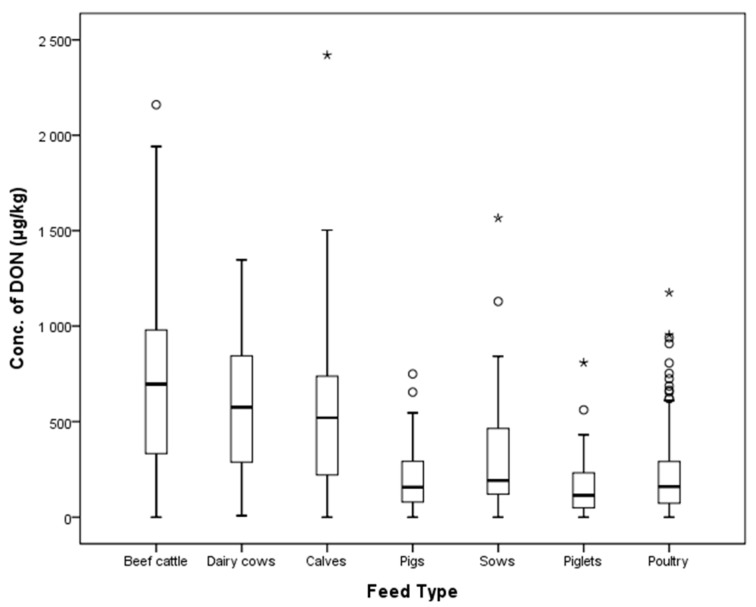
Distribution of DON in compound feeds (box-plot: whiskers at minimum and maximum, box at P25 and P75 with the line at P50; ° values above the 75th percentile plus 1.5 times the inter-quartile distance; * values above the 75th percentile plus 3.0 times the inter-quartile distance).

According to the EC, DON guidance level in feed for calves (<4 months), lambs, and kids is 2000 μg/kg and the guidance level for feed for pigs is 5000 μg/kg. In our study, no sample exceeded the EC guidance values for cattle and poultry feed. However, two compound feed samples (two gestating sow feed samples) out of 160 pig feed samples showed DON contamination in excess of the EC guidance value of 900 μg/kg. The distribution of DON according to type of livestock is shown in Figure 4.

**Figure 5:**
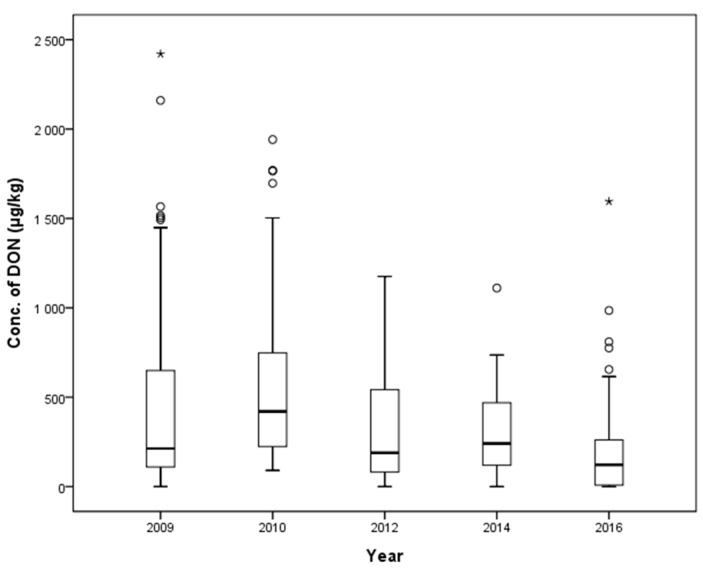
Distribution of DON in compound feed between 2009 and 2016 (box-plot: whiskers at minimum and maximum, box at P25 and P75 with the line at P50; ° values above the 75th percentile plus 1.5 times the inter-quartile distance; * values above the 75th percentile plus 3.0 times the inter-quartile distance).

A total of 494 compound feeds (128, 90, 150, 60, and 66 compound feeds from 2009, 2010, 2012, 2014, and 2016, respectively) were collected to analyze DON contamination levels (Figure 5). The mean contamination levels in each year were 426.8 (2009), 562.3 (2010), 314.5 (2012), 314.5 (2014), and 207.8 µg/kg (2016). The highest mean contamination level was found in 2010 (*p* < 0.001). There was a statistically-significant difference in mean contamination level for compound feeds collected in 2009, 2010, 2012, 2014, and 2016 in a one-way ANOVA (*p* < 0.001, Table 5), which revealed a generally decreasing trend without the contamination value in 2010. The occurrence of DON in selected years is shown in Figure 5.

### 2.3. Occurrence of DON in Feed Ingredients between 2009 and 2016

Feed ingredient samples (159 samples) were gathered at five time points: first in 2009 and then every other year from 2010 to 2016 (2010, 2012, 2014 and 2016). The samples consisted of 22 grains, 36 grain by-products including brans, 76 meals including vegetable proteins, 8 fibrous feeds, 13 food by-products, and 4 other feed ingredients including beans, seed nuts, and mixed formulation. Detailed data related to the DON contamination level in tested feed ingredient samples are summarized in Table 6.

**Figure 6:**
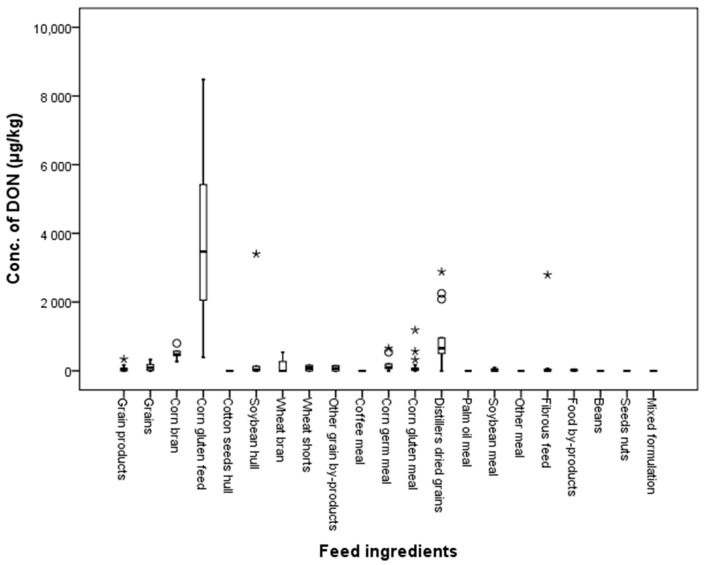
Distribution of DON in feed ingredients (box-plot: whiskers at minimum and maximum, box at P25 and P75 with the line at P50; ° values above the 75th percentile plus 1.5 times the inter-quartile distance; * values above the 75th percentile plus 3.0 times the inter-quartile distance).

The contamination rates with DON in feed ingredient samples were 72.7, 77.8, 61.8, 37.5 and 61.5% in grains, grain by-products, meals, fibrous feed, and food by-products, respectively. Over half of the collected feed ingredient samples (64%) showed contamination with DON. The average contamination level for DON was 555.3 μg/kg in the range of 0.01 to 8480.0 μg/kg. This mycotoxin was detected in grains, grain by-products, meals, fibrous feed, and food by-products at concentrations of 96.9, 1796.4, 240.8, 361.4 and 22.4 µg/kg, respectively. DON was not found in beans, seed nuts, or mixed formulation. The distribution of DON in feeds is shown in Figure 6.

According to the guidance values of the EC and South Korea for DON management (Table 1), DON in grains should be controlled at 8000 and 10,000 μg/kg, respectively. In this study, no samples exceeded these values. While the highest DON level (8480.0 μg/kg) was detected in corn bran, this did not exceed the EC guidance value for maize by-products (12,000 µg/kg).

**Figure 7:**
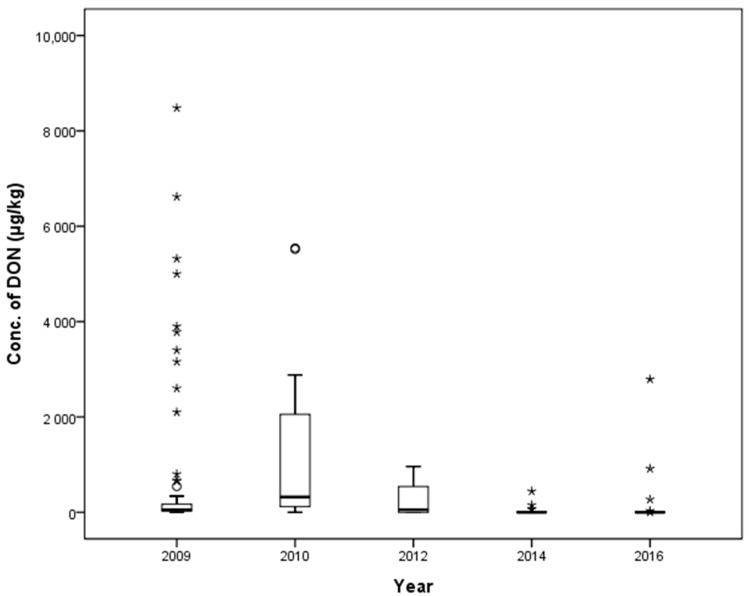
Distribution of DON in feed ingredients between 2009 and 2016 (box-plot: whiskers at minimum and maximum, box at P25 and P75 with the line at P50; ° values above the 75th percentile plus 1.5 times the inter-quartile distance; * values above the 75th percentile plus 3.0 times the inter-quartile distance).

A total of 159 feed ingredient samples were collected during the time points: 2009 (66), 2010 (23), 2012 (30), 2014 (17), and 2016 (23), and the mean contamination levels were 756.5, 1159.8, 231.8, 42.5, and 174.2 µg/kg, respectively. The highest mean contamination level was found in 2010 (*p* < 0.05). There was a statistically significant difference in mean contamination level for feed ingredients collected in 2009, 2010, 2012, 2014, and 2016 in a one-way ANOVA (*p* < 0.05, Table 7), which revealed a decreasing trend. The occurrence of DON in selected years is shown in Figure 7.

## 3. Discussion

The contamination of compound feeds by DON has been investigated in many countries. In Poland, DON was detected in 93.5% of 217 compound feed samples that were collected from 2006 to 2009 [24]. The average DON contamination level ranged from 136 to 225 µg/kg and with maximum contamination concentrations in the range of 409 to 2739 µg/kg. In South Africa, a total of 92 commercial compound feed samples were collected and 70.3% of the collected samples were contaminated with DON [25]. The mean DON contamination was 766.6 µg/kg, and the maximum value was 2352 µg/kg. Two swine feed samples exceeded the DON contamination limit level for South Africa (1000 µg/kg). In Kuwait, DON was found in 88.8% of poultry feed samples [26]. The mean DON concentration in poultry feeds was estimated at 261.1 µg/kg, within the range of 220 to 1200 µg/kg. In that study, the concentration of DON was found to be lower than EC-recommended levels. In Turkey, DON was detected in 48.3% of entire feed samples that ranged from 18.5 to 500 µg/kg [27]. The mean DON contamination level was analyzed as 59.8 µg/kg, and no sample exceeded the allowed level for DON in Turkey (5000 µg/kg for adult ruminants feed and 2000 µg/kg for lamb-calf feed). Another group reported a similar study that was conducted to determine DON levels in feed and feedstuff samples in Turkey [28]. A total of 106 samples were collected from several farms and feedstuffs manufacturers in Turkey. DON occurred in 18.4% of feedstuffs and 43.3% of feed samples. The highest determination level was 4769.6 μg/kg in maize gluten, indicating that DON content in feed and feedstuffs did not exceed the permitted levels. In 2016, Wu et al. [29] monitored the DON contamination in feed (2013–2015) obtained from several provinces in China. In that study, a total of 127 samples were analyzed using HPLC, and the highest detection level was at 3346.0 μg/kg in pig feed (pellet) in 2015, which exceeded the guidance values in pig complete feed in China (1000 μg/kg).

In this study, DON contamination was measured in 494 compound feed samples comprising 174 cattle feeds, 160 pig feeds, and 160 poultry feeds that were collected in 2009 and then every two years from 2010, 2012, 2014 and 2016. DON was detected in 95.3% of all of the compound feed samples with a range of 0.4 to 2420.0 µg/kg. DON was analyzed in 97.7% of cattle feeds, 93.1% of pig feeds, and 95.0% of poultry feeds. In the case of cattle and poultry feeds, the maximum contamination level was 2420.0 µg/kg and 1175.2 µg/kg, respectively. No sample exceeded the EU commission levels (5000 µg/kg for other feeds except calf and pig feeds). However, among pig feeds, two compound feeds including gestating sow feeds in 2009 (1566.0 µg/kg) and 2010 (1128.8 µg/kg) exceeded the EC guidance value (900 µg/kg for pig feeds).

Research related to DON contamination levels in feed ingredients has been performed in many countries. In Thailand, Poapolathep et al. monitored 90 wheat and wheat product samples [30] and showed that 18.9% of total samples were contaminated with DON in the range of 130 to 1130 µg/kg with a mean contamination level of 280.6 µg/kg, suggesting that the risk of DON exposure from wheat products is very low. In China, 83 feed ingredients samples were analyzed, and 95.2% of total samples were contaminated with DON with an average concentration of 1670.2 µg/kg [31]. The maximum contamination level of DON was 13,139.4 µg/kg, which exceeded the EC guidance value (8000 µg/kg). In the Netherlands, 140 maize silage samples and 20 wheat silage samples were collected, and DON was detected in 72% and 10% of the samples, respectively [32]. Average concentration levels of DON were 854 and 621 µg/kg, respectively, and maximum concentration levels were 3142 and 1165 µg/kg, respectively, and no samples exceeded the guidance value for DON (8000 µg/kg). In Tunisia, 83% of entire durum wheat samples were contaminated with DON [16], with an average concentration of DON that ranged from 12,800 ± 5% to 13,300 ± 13.3% µg/kg, which exceeded the EC guidance value for wheat (1750 µg/kg). In Serbia, a total of 289 feed ingredient samples were collected from 2004 to 2007 [33]. Some samples (33.2%) were contaminated with DON, and the average concentration of DON was 253 µg/kg. Three samples (two maize samples and a wheat sample) exceeded the guidance value. Wu et al. [29] estimated the level of contamination for DON in a total of 443 feed ingredient samples collected in China. Almost all samples were contaminated with DON (83.3% to 100%), and soybean meal showed the lowest incidence of DON (66.7%). Interestingly, in Hungary, wheat (305) and maize (108) were analyzed to estimate DON contamination levels collected from 2008 to 2015 [34]. In wheat samples, the highest mean contamination level of 2159 μg/mL and the lowest mean level of 181 μg/mL were observed in 2011 and 2012, respectively. However, in maize, the highest mean value (1261 μg/mL) and the lowest mean value (73 μg/mL) were observed in 2014 and 2009, respectively.

In this study, over half of the collected feed ingredient samples (64%) were found to be contaminated in South Korea. The average DON contamination concentration was 555.3 µg/kg with a range of 0.01 to 8480.0 µg/kg. DON was detected at a concentration of 96.9, 1796.4, 240.8, 361.4, and 22.4 µg/kg in grains, grain by-products, meals, fibrous feed, and food by-products, respectively. No samples exceeded the guidance values for the EU or South Korea (8000 and 1000 µg/kg, respectively). In the case of corn bran, a maximum of 8480.0 µg/kg of DON was detected, but this did not exceed the EC guidance value for corn bran (12,000 µg/kg).

There was a significant difference in the mean contamination level of DON in compound feed and feed ingredients over time in Korea that indicated a decreasing trend. This is mainly due to the continuous monitoring of *Fusarium* mycotoxins in feeds for many years and to the designation of guidance values for *Fusarium* mycotoxins since 2015.

## 4. Materials and Methods

### 4.1. Chemicals and Reagents

The standard reagent for DON analysis was purchased from Sigma Chemical Co. (St. Louis, MO, USA). Phosphate-buffered saline (PBS) was also obtained from Sigma-Aldrich for elution of DON in immunoaffinity column chromatography. Acetonitrile and methanol used in DON extraction were products of Honeywell Burdick & Jackson (Morris Plains, NJ, USA). The DONPREP kit (R-Biopharm^®^, Darmstadt, Germany) and DON Test kit (Vicam^®^, Palo Alto, CA, USA) were used for DON purification. The DON standard reagent was dissolved in acetonitrile to prepare standard solutions of high concentration, which were then diluted with 20% acetonitrile for use in analysis (acetonitrile: distilled water = 20:80, *v*:*v*).

### 4.2. Sampling of Feeds and Feed Ingredients

Contamination levels of DON were analyzed in 653 different feed samples (494 compound feed samples and 159 feed ingredients) produced in 2009 and every other year from 2010 to 2016. These samples were gathered at livestock feed factories from South Korea according to the annual procedure of the Ministry of Agriculture, Food, and Rural Affairs. The descriptions of compound feed and feed ingredient samples are shown in Table 8 and Table 9. All the samples were preprocessed according to the general guidelines on sampling from the FAO and WHO [35]. Random sample collection included choosing one kilogram per every ton of feed samples. Samples were collected four times from the same group, and the mixed sample was divided into another four groups. All of the divided samples were subdivided into 200 g and stored in a refrigerator at 4 °C. Detailed classification data for compound feed and feed ingredient samples are shown in the supplementary data (Tables S1–S5).

### 4.3. Extraction and Purification

The animal feed samples were ground to a particle size of 600 μm using a homogenizer, and 20 g of each feed was used as an analytical sample. The feed samples were mixed with distilled water (120 mL), and the mixture was extracted with a homogenizer at 7000 rpm for 2 min. After filtration of the extract through Whatman No. 4 filter paper (GE Healthcare Life Science, Maidstone, Kent, UK), 3 mL of the filtrate was added to an IAC prepared previously in a Vac Elut 20 Manifold (Agilent Technologies, Santa Clara, CA, USA). For an adequate reaction between the IAC packing material and DON, the flow rate was adjusted to 2 to 3 mL per minute. The extracts were passed through the IAC, and, after washing with 5 mL of distilled water, the distilled water was removed using a vacuum pump. The mycotoxin attached to the IAC was eluted with 3 mL methanol, which was slowly dropped under gravity. To increase the elution efficiency, back flushing was performed three times using a syringe before methanol was completely eluted from the IAC. The eluted solution was completely dried at 50 °C using a nitrogen micro-concentrator and re-dissolved in 20% acetonitrile. The re-dissolved solution was filtered through a syringe filter (0.22 μm pore size) and used as a solution for analyzing.

### 4.4. HPLC Analysis of DON

The concentration of DON in compound feed and feed ingredient samples was measured using HPLC. In analysis, Agilent 1100 series (Santa Clara, CA, USA) including a degasser, auto sampler, a ZORBAX Eclipse XDB-C18 column (4.6 × 250 mm, 5 μm), and a guard column C18 (4.6 × 10 mm, 5 μm) were used at 30 °C. DON was separated using HPLC for 20 min at a flow rate of 1 mL/min and detected with a diode array detector at 220 nm. The mobile phase was composed of HPLC grade water and acetonitrile, which was used in the gradient mode. The retention time was 4.4 min after injection of 20-μL samples.

### 4.5. Method Validation

The method of HPLC analysis for DON detection was verified by evaluating linearity, LOD, LOQ, accuracy, and precision. All parameters were calculated according to the ICH guidelines [36]. To determine linearity, the standard curve range was between 50 and 1000 μg/kg (50, 100, 200, 250, 500, and 1000 µg/kg), and the regression equation was calculated using the peak area and concentration of standard solution as parameters. The regression coefficient (*R*^2^) was used to confirm the linearity. The LOD and LOQ were calculated, which were the signal to noise ratio of 3 and 10. To determine accuracy, the recovery test included spiking a blank sample with various concentrations of DON standards, and the results are expressed as the recovery ratio. In this study, the precision indicated the degree of repeatability, and the percent relative standard deviation (%RSD) was used to calculate precision.

## Figures and Tables

**Table 1:** Recommended guidance values for DON in animal feed in the EU and South Korea.

Mycotoxin	Products Intended for Animal Feed	Guidance Value in mg/kg (ppm)	
		EU	Korea
**Deoxynivalenol**	Feed material		
	- Cereals and cereal products with the exception of maize by-products	8	10
	- Maize by-products	12	-
	Complementary and complete feed stuffs with the exception of maize by-products	5	-
	- Complementary and complete feed stuffs for pigs	0.9	0.9
	- Complementary and complete feed stuffs for calves (<4 months), lambs, and kids	2	-
	- Complementary and complete feed stuffs for ruminants	-	2
	- Other complementary and complete feed stuffs	-	5

**Table 2:** Summary of the method validation study.

*R* ^2^	LOD (µg/kg)	LOQ (µg/kg)	Recovery			
			Spiked Concentrations (µg/kg)	Mean Recovery (%)	SD	RSD (%)
0.999	1–10	3–35	50	95.6	13.3	13.9
			100	107.7	3.9	3.7
			200	95.4	7.7	4.0
			250	83.3	6.1	7.3
			500	108.3	4.3	3.9
			1000	88.8	12.4	1.4

*R*^2^, regression coefficient; LOD, limit of detection; LOQ, limit of quantitation; SD, standard deviation; RSD, relative standard deviation.

**Table 3:** DON levels in various compound types.

Livestock	Feed Type	*N* ^(a)^	LC (%) ^(b)^	Mean (μg/kg)	SD (μg/kg)
Beef cattle	Early beef cattle	21	0	646.9	452.6
	Middle beef cattle	8	12.5	709.2	473.3
	Late beef cattle	20	5	738.2	486.0
	Gestating beef cattle	29	0	831.2	521.3
	Lactating beef cattle	2	0	887.5	707.5
Dairy cows	Dairy cows in early lactation	16	0	505.2	350.3
	Dairy cows in mid lactation	5	0	801.4	296.7
	Dairy cows on dry	4	0	605.1	158.5
	High yielding dairy cows	9	0	601.8	375.6
	Gestating dairy cows	5	0	585.5	336.5
Calves	Early beef calves	9	11.1	580.3	256.0
	Middle beef calves	11	9.1	420.6	252.8
	Early dairy calves	1	0	125.0	0.0
	Middle dairy calves	15	0	600.6	387.4
	Late dairy calves	13	0	700.6	552.1
	Middle breeding calves	1	0	28.1	0.0
	Late breeding calves	5	0	315.0	213.3
Pigs	Early growing pigs	30	3.3	201.8	174.3
	Late growing pigs	18	0	217.6	167.8
Sows	Gestating sows	32	3.1	392.1	338.8
	Lactating sows	25	12	225.7	221.0
	Breeding gilts	1	0	162.8	0.0
Piglets	Sucking piglets	8	12.5	55.1	38.8
	Weanling piglets	46	10.9	179.7	164.5
Poultry	Early layer chicks	9	0	238.3	164.8
	Middle layer chicks	22	0	285.6	254.4
	Late layer chicks	8	0	406.2	377.3
	Early laying hens	27	7.4	314.7	258.7
	Middle laying hens	11	9.1	251.0	217.0
	Late laying hens	2	50	72.5	72.5
	Early broilers	37	2.7	148.6	135.5
	Late broilers	33	3.0	162.5	89.7
	Finishing broilers	2	0	152.8	34.8
	Breeding broilers	9	22.2	140.4	177.1

^(a)^ *N*, number of samples; ^(b)^ LC, percentage of left censored results (the percentage of data below the LOD) [23].

**Table 4:** DON concentration mean and differences in compound feeds.

Mycotoxins	Livestock	Conc. of Mycotoxin (μg/kg)		*F*	*p*
		Mean	SD		
Deoxynivalenol	Cattle	646.28 ^b^	452.58	94.363 ***	0.000
	Pig	231.47 ^a^	235.30		
	Poultry	221.93 ^a^	215.81		

^b^ > ^a^ = significant mean difference by Scheffe tests; *** *p* < 0.001; *F*, *F*-value.

**Table 5:** The mean values of DON concentration and differences in compound feeds across the years.

Mycotoxin	Year	Conc. of Mycotoxin (μg/kg)		*F*	*p*
		Mean	SD		
Deoxynivalenol	2009	426.76 ^b,c^	465.27	11.434 ***	0.000
	2010	562.34 ^c^	432.66		
	2012	314.49 ^a,b^	292.86		
	2014	314.5 ^a,b^	230.09		
	2016	207.83 ^a^	286.21		

^c^ > ^b^, ^b^ > ^a^ = significant mean difference by Scheffe tests, *** *p* < 0.001; *F*, *F*-value.

**Table 6:** DON concentrations in feed ingredients.

Class	Feed Groups	*N* ^(a)^	LC (%) ^(b)^	Mean (μg/kg)	SD (μg/kg)
Grains	Grains	12	16.7	115.9	107.0
	Grain products	10	40.0	74.2	102.2
Grain byproducts (brans)	Corn gluten feed	16	0	3603.7	2268.2
	Soybean hull	6	33.3	600.1	1252.9
	Wheat shorts	2	50.0	81.0	81.0
	Cotton seeds hull	2	100.0	0.0	0.0
	Wheat bran	3	66.7	180.4	255.2
	Corn bran	5	0	511.4	174.8
	Other grain byproducts	2	50.0	75.4	75.4
Meal	Soybean meal	16	50.0	27.1	32.3
	Corn gluten meal	22	18.2	134.8	261.7
	Corn germ meal	12	16.7	182.4	198.3
	Distillers dried grains	14	21.4	908.1	850.7
	Coffee meal	1	100.0	0.0	0.0
	Palm oil meal	7	100.0	0.0	0.0
	Other meal	4	100.0	0.0	0.0
Fibrous feed	Fibrous feed	8	62.5	361.4	918.2
Food byproducts	Food byproducts	13	38.5	22.4	19.2
Beans	Beans	1	100.0	0.0	0.0
Seeds nuts	Seeds nuts	2	100.0	0.0	0.0
Mixed formulation	Mixed formulation	1	100.0	0.0	0.0

^(a)^ *N*, number of samples; ^(b)^ LC, percentage of left censored results (the percentage of data below the LOD) [23].

**Table 7:** The mean DON concentration and differences in feed ingredients across years.

Mycotoxin	Year	Conc. of Mycotoxin (μg/kg)		*F*	*p*
		Mean	SD		
Deoxynivalenol	2009	756.51	1735.03	3.198 *	0.015
	2010	1159.80	1636.07		
	2012	231.77	289.41		
	2014	42.49	110.32		
	2016	174.19	602.91		

* *p* < 0.05; *F*, *F*-value.

**Table 8:** Compound feed samples between 2009 and 2016.

Livestock	Feed Type	No. of Samples					
		Total	2009	2010	2012	2014	2016
Beef cattle	Early beef cattle	21	9	2	6	2	2
	Middle beef cattle	8	-	3	-	2	3
	Late beef cattle	20	7	4	6	1	2
	Gestating beef cattle	29	13	7	5	2	2
	Lactating beef cattle	2	-	-	-	-	2
Dairy cows	Dairy cows in early lactation	16	6	2	6	1	1
	Dairy cows in mid lactation	5	2	2	-	1	-
	Dairy cows on dry	4	1	-	-	2	1
	High yielding dairy cows	9	1	-	5	2	1
	Gestating dairy cows	5	-	3	-	1	1
Calves	Early beef calves	9	-	-	5	1	3
	Middle beef calves	11	1	-	6	1	3
	Early dairy calves	1	-	-	-	-	1
	Middle dairy calves	15	4	3	6	1	1
	Late dairy calves	13	3	4	5	1	-
	Middle breeding calves	1	-	-	-	-	1
	Late breeding calves	5	1	-	-	2	2
Pigs	Early growing pigs	30	12	5	10	3	-
	Late growing pigs	18	4	5	5	4	-
Sows	Gestating sows	32	6	10	10	3	3
	Lactating sows	25	5	5	10	3	2
	Breeding gilts	1	-	-	-	1	-
Piglets	Sucking piglets	8	-	-	5	2	1
	Weanling piglets	46	13	5	10	4	14
Poultry	Early layer chicks	9	3	3	-	1	2
	Middle layer chicks	22	4	7	6	3	2
	Late layer chicks	8	-	-	5	3	-
	Early laying hens	27	5	6	10	3	3
	Middle laying hens	11	-	-	5	3	3
	Late laying hens	2	-	-	-	-	2
	Early broilers	37	12	7	10	4	4
	Late broilers	33	12	7	9	3	2
	Finishing broilers	2	-	-	-	-	2
	Breeding broilers	9	4	-	5	-	-
Total		494	128	90	150	60	66

**Table 9:** Feed ingredient samples between 2009 and 2016.

Class	Feed Type	No. of Samples					
		Total	2009	2010	2012	2014	2016
Grain	Grain	12	7	4	-	-	1
	Grain products	10	7	-	2	-	1
Grain-by products (bran)	Corn gluten feed	16	9	5	2	-	-
	Soybean hull	6	5	-	-	1	-
	Wheat shorts	2	-	-	1	1	-
	Cotton seeds hull	2	-	-	1	1	-
	Wheat bran	3	-	-	2	1	-
	Corn bran	5	1	-	2	1	1
	Other grain by-products	2	-	-	-	1	1
Meal (vegetable proteins)	Soybean meal	16	10	-	2	3	1
	Corn gluten meal	22	10	5	4	3	-
	Corn germ meal	12	4	4	2	1	1
	Distillers dried grains	14	2	5	6	-	1
	Coffee meal	1	-	-	1	-	-
	Palm oil meal	7	4	-	3	-	-
	Other meal	4	-	-	-	1	3
Fibrous feed	Fibrous feed	8	-	-	-	2	6
Food by-products	Food by-products	13	7	-	2	1	3
Beans	Beans	1	-	-	-	-	1
Seeds nuts	Seeds nuts	2	-	-	-	-	2
Mixed formulation	Mixed formulation	1	-	-	-	-	1
Total		159	66	23	30	17	23

## Supplementary Materials

The following are available online at http://www.mdpi.com/2072-6651/10/3/127/s1. Table S1: Description of compound feeds for cattle. Table S2: Description of compound feeds for swine. Table S3: Description of compound feeds for poultry. Table S4: Description of compound feeds for dairy cows. Table S5: Description of feed ingredients.
