# Supplementary material for: Long-Term Occurrence of Deoxynivalenol in Feed and Feed Raw Materials with a Special Focus on South Korea
Source: Toxins (Basel). 2018 Mar 16;10(3):127. doi: 10.3390/toxins10030127 (PMC5869415; doi:10.3390/toxins10030127)
Supplement: Supplementary file 1 [file toxins-10-00127-s001.pdf]

Supplementary Materials: Long-Term Occurrence of Deoxynivalenol in Feed and Feed Raw Materials with a Special Focus on South Korea

Juhee Park, Hansub Chang, Dongho Kim, Soohyun Chung and Chan Lee

Table S1. Description of compound feeds for cattle.

Feed Type	Period for feeding
Early breeding calves	Birth to 3 months
Middle breeding calves	3 to 6 months
Late breeding calves	6 to 12 months
Sire or breeding bull	After 13 months
Gestating beef	13 months to farrowing
Lactating beef	Farrowing to 3 months after birth
Early beef calves	Before 3 months
Middle beef calves	After 3 months to 250 kg of body weight
Early beef cattle	250 kg to 400 kg of body weight
Middle beef cattle	400 kg to 500 kg of body weight
Late beef cattle	Over 500 kg of body weight

Table S2. Description of compound feeds for swine.

Feed Type	Period for feeding
Sucking piglets	Before lactating
Weanling piglets	Over 5 kg of body weight or after lactating to 20 kg of body weight
Early growing pigs	20 kg to 50 kg of body weight
Late growing pigs	50 kg to 80 kg of body weight
Growing pigs	50 kg or 80 kg of body weight to before the 15th for shipment
Growing-finishing pigs	Before the 15th for shipment to shipment
Sire, boar	Over 25 kg of body weight
Gilts	25 kg of body weight to before pregnant
Gestating sows	Gestating period
Lactating sows	Lactating period

Table S3. Description of compound feeds for poultry.

Feed Type	Period for feeding
Early layer chicks	Before 6 to 10 weeks
Middle layer chicks	6 to 12 weeks or 2 weeks before laying
Late layer chicks	12 weeks to 2 weeks before laying
Early broiler chicks	3 weeks or before 6 weeks
Middle broiler chicks	3 weeks or 6 weeks to before laying
Before laying	2 weeks before laying to laying commencement
Early laying	Laying commencement to 40 weeks
Middle laying	40 weeks to 65 weeks
Late laying	After 65 weeks
Breeding broiler	Layer breeder or broiler breeder
Early broiler	Before 3 weeks
Middle broiler	3 weeks to before the 7–10th before shipment
Finishing broiler	After the 7–10th before shipment to shipment

Table S4. Description of compound feeds for dairy cows.

Feed Type	Period for feeding
Early dairy calves	Birth to 3 months
Middle dairy calves	3 months to 6 months
Late dairy calves	6 months to before pregnancy
Gestating dairy cows	Pregnancy to 2 months after farrowing
Dairy sires	After 13 months
Dairy cows in early lactation	After farrowing to lactation 3 months (31 to 40 kg of milk production)
Dairy cows in mid lactation	Lactation 3 months to 6 months (21–30 kg of milk production)
Dairy cows in late lactation	Lactation 6 months to dry period (11–20 kg of milk production)
Dairy cow on dry	Dry period (Less than 10 kg of milk production)
High yielding dairy cows	Over 40 kg of milk production

Table S5. Description of feed ingredients.

Class	Feed type
Grains	Grains<br>Grain products
Grain by-products (Bran)	Corn gluten feed<br>Soybean hull<br>Wheat shorts<br>Cotton seeds hull<br>Wheat bran<br>Corn bran<br>Other grain by-products
Meal (Vegetable proteins)	Soybean meal<br>Wheat gluten<br>Corn gluten meal<br>Corn germ meal<br>Distillers dried grains<br>Coffee meal<br>Palm oil meal<br>Other meal
Fibrous feed	Fibrous feed
Food by-products	Food by-products
Beans	Beans
Seed nuts	Seed nuts
Mixed formulation	Mixed formulation
